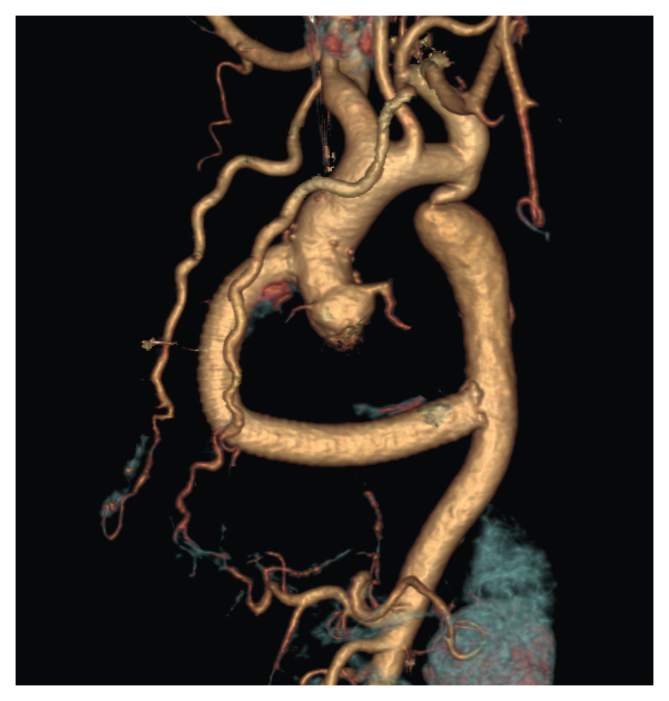# Ascending to Descending Aortic Bypass for Complex Aortic Coarctation

**DOI:** 10.1016/j.ejvsvf.2022.12.001

**Published:** 2022-12-20

**Authors:** Julio Ellacuriaga San Martin, Christian Dinges

**Affiliations:** Paracelsus Medical University Salzburg, Salzburg, Austria

A 42 year old obese man with severe aortic regurgitation associated with bicuspid aortic valve and left ventricular dilatation presented with a hypertensive crisis (blood pressure [BP] 228/117 mmHg) and chest pain. Physical examination showed an ankle brachial index (ABI) of 0.6 and absent femoral pulses in both lower limbs. Computed tomography angiography showed an ascending aortic diameter of 4.7 cm and aortic coarctation with a critically stenosed aorta and prominent internal mammary arteries providing the collateral circulation. With extracorporeal circulation, reconstruction of the bicuspid aortic valve and the aortic root was done by means of the hemi-Yacoub technique and extra-aortic annuloplasty using a Dacron ring, as well as plication of the fused cusp. Moreover, an ascending to descending aortic bypass with a 19 mm expanded polytetrafluoroethylene graft was inserted after cleavage of the dorsal pericardium. The post-operative course was uneventful. The patient recovered distal pulses and had a normal ABI of 1.0 bilaterally. The patient had improvement in his hypertension (BP 133/81 mmHg) and reduced requirements for antihypertensive medications.

**Figure:**